# Metallic Materials: Structure Transition, Processing, Characterization and Applications (Second Edition)

**DOI:** 10.3390/ma19163403

**Published:** 2026-08-11

**Authors:** Jing Hu

**Affiliations:** 1 Jiangsu Key Laboratory of Materials Surface Science and Technology, Changzhou University, Changzhou 213164, China; jinghoo@126.com; Tel.: +86-0519-86330095; 2 Huaide College, Changzhou University, Jingjiang 214500, China

The field of metallic materials is experiencing a paradigm shift, driven by the need for higher performance, sustainability, and efficiency. Recent developments span several key areas, which are listed as follows: (1) Advanced Processing and Surface Engineering: Significant progress has been made in novel heat treatment and surface modification technologies (e.g., plasma nitriding, post-oxidation) to enhance the wear resistance, hardness, and corrosion resistance of both ferrous and non-ferrous metals [1,2,3,4,5]. (2) Data-Driven Discovery: Machine learning (ML) and deep learning (DL) are revolutionizing the field by enabling high-throughput microstructure quantification, multi-modal property prediction, and inverse design for novel alloys, moving away from traditional “trial-and-error” approaches [6,7,8,9,10]. (3) Additive Manufacturing (AM): AM techniques, particularly for high-performance nickel superalloys and functionally graded materials, are being refined to overcome challenges such as microstructure heterogeneity, cracking, and residual stresses [11,12,13,14].

Despite these advances, the following critical knowledge gaps remain: (1) Data Scarcity and Model Generalization: The lack of standardized, high-quality datasets limits the robustness and generalization of ML models, which often fail when applied outside their narrow training parameters [15,16,17,18]. (2) Bridging Scales and Mechanisms: Integrating atomic-scale descriptors with macroscopic performance (e.g., fatigue life, high-temperature behavior) remains a formidable challenge, requiring more sophisticated multi-scale modeling frameworks [19,20,21]. (3) Reliability and Interpretability: The “black-box” nature of many ML models hinders their physical interpretability and reliability, slowing their adoption in critical, high-consequence industrial applications [22,23,24].

This Special Issue addresses these challenges by presenting cutting-edge research from leading researchers and practitioners that exemplifies the integration of advanced characterization, novel processing, and data-driven analysis on the topic “Metallic Materials: Structure Transition, Processing, Characterization and Applications”.

The contributions encompass original scientific work spanning fundamental research and application-oriented studies. They address the identified knowledge gaps through the following key directions: (1) Demonstrating Novel Processing–Microstructure–Performance Pathways: The included studies provide direct experimental evidence of how novel and complex treatments (e.g., annealing–sandblasting on welded titanium, pulsed magnetic fields on aluminum bronze) can effectively regulate microstructure to solve specific industrial problems, such as the adverse impact of welds on product quality. (2) Advancing Microstructural Characterization: By exploring detailed precipitation sequences in steels and the local structure of complex niobates, this Special Issue provides critical, fundamental data needed to build more reliable structure–property relationships. (3) Showcasing the Application of ML: One of the papers explicitly demonstrates the use of an optimized artificial neural network to predict the flow stress of In718 alloys at high temperatures, bridging the gap between advanced computational tools and practical materials processing.

By collating these diverse yet interconnected studies, this Special Issue serves as a comprehensive resource that validates new methodologies and provides crucial high-quality experimental data, which is essential for training and validating the next generation of data-driven models.

The synergy between advanced experimental work and computational modeling highlighted in this Special Issue points towards several promising future research directions: (1) Physics-Informed Machine Learning: Future models must integrate physical principles and mechanistic knowledge to overcome data scarcity and enhance interpretability. This will yield more reliable and trustworthy predictions for complex metallic systems. (2) Autonomous and Closed-Loop Materials Discovery: The ultimate goal is to create integrated platforms that combine AI, in situ experiments, and autonomous characterization to close the loop between process control, structural realization, and performance validation, enabling faster alloy development cycles. (3) Digital Twins for Manufacturing: Developing digital twin frameworks for processes such as additive manufacturing and heat treatment will be crucial for predicting microstructure evolution and final properties, optimizing processes in real time, and reducing experimental trial costs. (4) High-Throughput and Multi-Modal Characterization: To provide the vast, high-quality spatial datasets needed for ML, future research must focus on developing and standardizing high-throughput, multi-modal characterization techniques capable of capturing complex heterogeneities across multiple length scales.

There are seven research papers published in this Special Issue, the website of which has already received 11,169 visits, which implies that these papers demonstrate exceptional academic value and significant impact. In order to express the high quality of the efforts and progress that these outstanding authors have made, the seven research papers are briefly summarized below.

Maurya et al. [25] contributed a paper entitled “Artificial Neural Network Modeling of Ti-6Al-4V Alloys to Correlate Their Microstructure and Mechanical Properties”, which proposed an artificial neural network (ANN) to understand the relationship between microstructural features and mechanical properties by varying the heat treatment processes of Ti-6Al-4V alloy.

Yu et al. [26] contributed a paper entitled “Dynamic Recrystallization Model of High-Temperature Deformation and Finite Element Analysis of Microstructure Evolution of 14Cr1Mo Pressure Vessel Steel”, which established a dynamic recrystallization (DRX) kinetic model and grain size model of 14Cr1Mo steel. The results showed excellent agreement between the simulated and experimentally measured grain sizes, confirming the high accuracy of the dynamic recrystallization models. These models provide a theoretical basis for finite element simulation and microstructure control in the manufacturing of super-large pressure vessel tube sheet forgings.

Zhang et al. [27] contributed a paper entitled “Effect of Heat Treatment on Microstructures and Mechanical Properties of TC4 Alloys Prepared by Selective Laser Melting”, which proposed that appropriate heat treatment could improve the microstructures and properties of selective laser melting (SLM)-fabricated TC4 alloys. After heat treatment, the ultimate tensile strength slightly decreases, but the elongation significantly increases, and the fracture mechanism shifts from mixed brittle–ductile fracture to ductile fracture. This work provides a theoretical basis for improving the microstructures and properties of SLM-fabricated TC4 alloys through heat treatment.

Hu et al. [28] contributed a paper entitled “Analysis of Crack Cause of Parking Ratchet during the Manufacturing Process”, which proposed a systematic analysis of the root cause of parking ratchet cracking, and provided a way to avoid such failure. This study provides a feasible direction for the failure analysis and control of cracks on parking ratchets during the manufacturing process.

Yang et al. [29] contributed a paper entitled “In Situ-Reinforced Phase Evolution and Mechanical Properties of CoCrFeNi High-Entropy Alloy Composite Coating on Q235B by Laser Cladding with Nb Addition”. In this work, laser cladding (LC) technology was applied to deposit a CoCrFeNi high-entropy alloy (HEA) composite coating—with varying proportions of tungsten carbide (WC) and Nb—onto a Q235B substrate. The aim was to enhance the wear and corrosion resistance of the substrate.

Yang et al. [30] contributed a paper entitled “Relationship between Texture, Hydrogen Content, Residual Stress and Corrosion Resistance of Electrodeposited Chromium Coating: Influence of Heat Treatment”. In this work, the effect of post-heat-treatment processes on the relationship between texture, hydrogen content, residual stress, and corrosion resistance of hexavalent chromium (Cr(VI)) coatings deposited on Cr–Ni–Mo–V steel substrates was systematically investigated. A valuable conclusion was drawn: for future assessments of heat treatment effects on coating properties, the reasonableness of the process can be determined to a certain extent solely by measuring the macrotexture.

Xu et al. [31] contributed a paper entitled “The Effect of Different Thermomechanical Treatments on the Metastable Phase in a Cu-Ni-Be Alloy”. This study primarily investigated the microstructural and mechanical properties of Cu-Ni-Be alloys subjected to thermomechanical treatments at 30% and 75% deformation levels. No prominent cellular structures were observed at either deformation level.

We extend our sincere gratitude to all the authors who contributed to this Special Issue. Their significant research contributions made this Special Issue valuable, and we genuinely appreciate all the efforts they made.

In conclusion, we hope that this Special Issue serves as a comprehensive resource, fostering innovation and dialog among researchers and industry experts in this dynamic field.

For further information, readers are encouraged to refer to the complete papers.

## References

[1] Tarnowski M., Borowski T., Skrzypek S., Kulikowski K., Wierzchoń T. (2021). Shaping the structure and properties of titanium and Ti6Al7Nb titanium alloy in low-temperature plasma nitriding processes. J. Alloys Compd..

[2] Kang Q., Wei K., Fan H., Liu X., Hu J. (2022). Ultra-high efficient novel plasma aluminum-nitriding methodology and performances analysis. Scr. Mater..

[3] Li L., Liu R., Liu Q., Wu Z., Meng X., Fang Y. (2022). Effects of Initial Microstructure on the Low-Temperature Plasma Nitriding of Ferritic Stainless Steel. Coatings.

[4] Shen J., Wei K., Liu X., Hu J. (2024). Regulation of phase partition and wear resistance for FeCoCrV high entropy alloy by heat treatment. Intermetallics.

[5] Ni J., Ma H., Wei W., An X., Yu M., Hu J. (2024). Novel Effect of Post-Oxidation on the Comprehensive Performance of Plasma Nitriding Layer. Coatings.

[6] Sheng Y., Qin X., Wang Q., Yang H. (2026). Deep learning-driven innovation in metallic materials: A comprehensive review on microstructure analysis, property prediction, and inverse design. J. Mater. Sci. Technol..

[7] Cheng Z., Zhou J., Guo G., Chen S., Ye X., Wei X., Zhang Y., Zhang Z. (2025). Machine learning-based investigation of δ phase precipitation behavior in IN718 alloy and its influence on tensile properties. Mater. Sci. Eng. A.

[8] Mathieu C., Chris B., Anjaria D., Park H., Wang H., Vecchio K., Stinville J. (2025). Learning metal microstructural heterogeneity through spatial mapping of diffraction latent space features. npj Comput. Mater..

[9] Miao B., Lin G., Zhang Y., Cheng Y., Yang H. (2026). Machine learning applications in metallic materials: Recent advances and future perspectives. J. Alloys Compd..

[10] Iman P., Salim B., Mercuri F., Fantuzzi N., Dehghani H., Izadi R., Ibrahim H., Lengiewicz J., Belouettar-Mathis M. (2025). Artificial intelligence in materials science and engineering: Current landscape, key challenges, and future trajectories. Compos. Struct..

[11] Hakami A., Ojo S.A., Abere D., Uzuh F.D., Robert R. (2025). Advancements in metal additive manufacturing: Opportunities, limitations, impact on properties, and potential solutions: A review. Prog. Addit. Manuf..

[12] Kumar M.D.B., Madesh R., Adarsh S.J. (2026). Recent developments and future perspectives in metal additive manufacturing: Techniques, challenges, and functional performance. J. Alloys Compd..

[13] Ojo S.A., Abere D.V. (2025). Recent advances in surface modification of metals and alloys: Enhancing material protection and performance in challenging environments. Int. J. Adv. Manuf. Technol..

[14] Gogulraj G., Rajamurugan G. (2025). A review on additive manufacturing of dissimilar metals: Current challenges, industrial applications, and AI/ML integration strategies. Phys. Scr..

[15] Bae J., Seol J., Moon J., Sohn S., Jang M., Um H., Lee B., Kim H. (2018). Exceptional phase-transformation strengthening of ferrous medium-entropy alloys at cryogenic temperatures. Acta Mater..

[16] Zhao S., Liu M., Tian J., Dai F., Xu G. (2024). Review on Heat Treatment and Surface Modification Technology of High-Strength Bainite Steels. Steel Res. Int..

[17] Nixon M., Lebensohn R., Cazacu O., Liu C. (2010). Experimental and finite-element analysis of the anisotropic response of high-purity α-titanium in bending. Acta Mater..

[18] Hiremath P., Sharma S., Gowrishankar M., Shettar M., Gurumurthy B. (2020). Effect of post carburizing treatments on residual stress distribution in plain carbon and alloy steels-a numerical analysis. J. Mater. Res. Technol..

[19] Salawu E., Adediran A., Ajayi O., Inegbenebor A., Dirisu J. (2022). On the analyses of carbon atom diffused into grey cast iron during carburisation process. Sci. Rep..

[20] Nishimoto A., Fukube T., Maruyama T. (2019). Microstructural, mechanical, and corrosion properties of plasma-nitrided CoCrFeMnNi high-entropy alloys. Surf. Coat. Technol..

[21] Egea A., Rodriguez A., Celentano D., Calleja A., De Lacalle L. (2019). Joining metrics enhancement when combining FSW and ball-burnishing in a 2050 aluminium alloy. Surf. Coat. Technol..

[22] Zhao Y., Ni J., Qi L., Hu J., An X., Wei W. (2026). Effect of Cyclic Post-oxidation on the Characteristics and Properties of Plasma-Nitrided Layer. Vacuum.

[23] Unal O., Maleki E., Varol R. (2021). Comprehensive analysis of pulsed plasma nitriding preconditions on the fatigue behavior of AISI 304 austenitic stainless steel. Int. J. Miner. Metall. Mater..

[24] Kovács D., Quintana I., Dobránszky J. (2019). Effects of different variants of plasma nitriding on the properties of the nitrided layer. J. Mater. Eng. Perform..

[25] Maurya A., Narayana P., Yeom J., Hong J. (2025). Artificial Neural Network Modeling of Ti-6Al-4V Alloys to Correlate Their Microstructure and Mechanical Properties. Materials.

[26] Yu B., Zhang B., Shi R., Mao F. (2025). Dynamic Recrystallization Model of High-Temperature Deformation and Finite Element Analysis of Microstructure Evolution of 14Cr1Mo Pressure Vessel Steel. Materials.

[27] Zhang J., Shi Y., Shen S., Zhang S., Ding H., Pan X. (2025). Effect of Heat Treatment on Microstructures and Mechanical Properties of TC4 Alloys Prepared by Selective Laser Melting. Materials.

[28] Fan H., Xie X., Hu J., Wang D. (2025). Analysis of Crack Cause of Parking Ratchet During the Manufacturing Process. Materials.

[29] Yang F., Xiao Z., Chen Z., Jin H., Gao C., Huang J. (2025). In Situ-Reinforced Phase Evolution and Mechanical Properties of CoCrFeNi High-Entropy Alloy Composite Coating on Q235B by Laser Cladding with Nb Addition. Materials.

[30] Yang J., Ji P., Yang X., Wu L., Ding X., Zhang J., Lian Y., Dou S. (2024). Relationship between Texture, Hydrogen Content, Residual Stress and Corrosion Resistance of Electrodeposited Chromium Coating: Influence of Heat Treatment. Materials.

[31] Xu J., Yuan Q., Jia J., Wang T., Shen Y., Zhu Z. (2025). The Effect of Different Thermomechanical Treatments on the Metastable Phase in a Cu-Ni-Be Alloy. Materials.

